# The beneficial effect of alpha-blockers for ureteral stent-related discomfort: systematic review and network meta-analysis for alfuzosin versus tamsulosin versus placebo

**DOI:** 10.1186/s12894-015-0050-5

**Published:** 2015-06-24

**Authors:** Jong Kyou Kwon, Kang Su Cho, Cheol Kyu Oh, Dong Hyuk Kang, Hyungmin Lee, Won Sik Ham, Young Deuk Choi, Joo Yong Lee

**Affiliations:** Department of Urology, Haeundae Paik Hospital, Inje University College of Medicine, Busan, South Korea; Department of Urology, Gangnam Severance Hospital, Urological Science Institute, Yonsei University College of Medicine, Seoul, South Korea; Department of Urology, Yangpyeong Health Center, Yangpyeong, South Korea; Division of Epidemic Intelligence Service, Korea Centers for Disease Control and Prevention, Osong, South Korea; Department of Urology, Severance Hospital, Urological Science Institute, Yonsei University College of Medicine, 50-1 Yonsei-ro, Seodaemun-gu Seoul, 120-752 South Korea

**Keywords:** Ureter, Stents, Adrenergic alpha-antagonists, Meta-analysis, Bayes theorem

## Abstract

**Background:**

This study was carried out a network meta-analysis of evidence from randomized controlled trials (RCTs) to evaluate stent-related discomfort in patients with alfuzosin or tamsulosin versus placebo.

**Methods:**

Relevant RCTs were identified from electronic databases. The proceedings of appropriate meetings were also searched. Seven articles on the basis of RCTs were included in our meta-analysis. Using pairwise and network meta-analyses, comparisons were made by qualitative and quantitative syntheses. Evaluation was performed with the Ureteric Stent Symptoms Questionnaire to assess the urinary symptom score (USS) and body pain score (BPS).

**Results:**

One of the seven RCTs was at moderate risk of bias for all quality criteria; two studies had a high risk of bias. In the network meta-analysis, both alfuzosin (mean difference [MD];−4.85, 95 % confidence interval [CI];−8.53–−1.33) and tamsulosin (MD;−8.84, 95 % CI;−13.08–−4.31) showed lower scores compared with placebo; however, the difference in USS for alfuzosin versus tamsulosin was not significant (MD; 3.99, 95 % CI;−1.23–9.04). Alfuzosin (MD;−5.71, 95 % CI;−11.32–−0.52) and tamsulosin (MD;−7.77, 95 % CI;−13.68–−2.14) showed lower scores for BPS compared with placebo; however, the MD between alfuzosin and tamsulosin was not significant (MD; 2.12, 95 % CI;−4.62–8.72). In the rank-probability test, tamsulosin ranked highest for USS and BPS, and alfuzosin was second.

**Conclusion:**

The alpha-blockers significantly decreased USS and BPS in comparison with placebo. Tamsulosin might be more effective than alfuzosin.

## Background

In 1978, the ureteral double-J stent was first described by Finney et al. [1, 2]. Ureteral double-J stent insertion has been one of the most common urologic procedures; however, indwelling stents are often accompanied by significant morbidity including voiding and storage symptoms, flank pain, hematuria, and infection [3]. Symptoms of stent discomfort, including bladder irritation symptoms and flank pain or discomfort, are generally treated with oral analgesics, such as narcotics and anti­inflammatory medications; however these medications are only moderately effective. Alpha-blockers alleviate bladder irritation due to stents, resulting in reduced incidence of dysuria, frequency, and pain compared to placebo [4]. Ureteral stent discomfort may be due to spasms of the ureteral smooth muscle that surrounds the indwelling foreign object and may run the length of the ureter. Further, irritation of the trigone, which also has alpha-1d receptors, may be caused by the intravesical lower coil of the stent. Alternatively, voiding may increase pressure on the renal pelvis and cause discomfort [5]. Several studies have investigated if alpha-blockers can alleviate symptoms related to stent placement [6]. In 2011, Lamb et al. reported a pair-wise meta-analysis of randomized controlled trials (RCTs) indicating that orally administrated alpha-blockers reduce stent-related discomfort and storage symptoms as evaluated by the Ureteric Stent Symptoms Questionnaire (USSQ) [7].

Newly introduced network meta-analysis is a meta-analysis in which multiple treatments are compared using direct comparisons of interventions within RCTs and indirect comparisons across trials based on a common comparator [8–10]. The present systematic review and network meta-analysis examined available RCTs to study the effects of alpha-blockers on stent-related symptoms.

## Methods

### Inclusion criteria

Published RCTs that accorded with the following criteria were included. (i) The design of study had an assessment for alpha-blockers to treat ureteral stent discomfort. (ii) A match was performed between the baseline characteristics of patients from two groups, including the total number of subjects and the values of each index. (iii) Alpha-blockers were analyzed with standard therapy or a placebo group. (iv) Standard indications for ureteral stenting, such as stone treatment, ureteroscopic procedures, and ureteral surgery including pyeloplasty, were accepted. (v) Endpoint outcome parameters were described using USSQ, including urinary symptom score (USS) and body pain score (BPS). (vi) The full text of the study was available in English. This report was prepared in compliance with the Preferred Reporting Items for Systematic Reviews and Meta-Analyses (PRISMA) statement (accessible at http://www.prisma-statement.org/).

### Search strategy

A literature search of all publications before 31 January 2014 was performed in EMBASE and PubMed. Additionally, a cross-reference search of eligible articles was performed to check studies that were not found during the computerized search. Combinations of the following MeSH terms and keywords were used: tamsulosin, alfuzosin, doxazosin, terazosin, silodosin, prazosin, alpha, stent, discomfort, pain, complication, ureter, ureteral, ureteric, and randomized controlled trial.

### Extraction of data

A researcher (JKK) screened all titles and abstracts identified by the search strategy. Other two researchers (JYL and DHK) independently evaluated the full text of each paper to determine whether a paper met the inclusion criteria. Disagreements were resolved by discussion until a consensus was reached or by arbitration mediated by another researcher (KSC).

### Quality assessment for each study

After the final group of papers was agreed on, two researchers (JYL and JKK) independently evaluated the quality of each article. The Cochrane’s risk of bias as a quality assessment tool for RCTs were used. The assessment included assigning a judgment of “yes,” “no,” or “unclear” for each domain to designate a low, high, or unclear risk of bias, respectively. If one or no domain was deemed “unclear” or “no,” the study was classified as having a low risk of bias. If four or more domains were deemed “unclear” or “no,” the study was classified as having a high risk of bias. If two or three domains were deemed “unclear” or “no,” the study was classified as having a moderate risk of bias [11]. Quality assessment was performed with Review Manager 5 (RevMan 5.2.11, Cochrane Collaboration, Oxford, UK).

### Heterogeneity tests

Heterogeneity on included studies was examinated using the Q statistic and Higgins’ I^2^ statistic [12]. Higgins’ I^2^ measures the percentage of total variation due to heterogeneity rather than chance across studies. Higgins’ I^2^ was calculated as follows:

$$ {\mathrm{I}}^2=\frac{\mathrm{Q}\hbox{-} \mathrm{d}\mathrm{f}}{\mathrm{Q}}\times 100\%, $$

in which “Q” was Cochran’s heterogeneity statistic, and “df” was the degrees of freedom.

An I^2^ ≥ 50 % was considered to represent substantial heterogeneity. For the Q statistic, heterogeneity was deemed to be significant for p less than 0.10 [13]. If there was evidence of heterogeneity, the data were analyzed using a random-effects model. A summary estimate of the test sensitivity was obtained with 95 % confidence intervals (CIs) after secondary examination of heterogeneity in the random-effects model using radial plots [14]. Studies in which positive results were confirmed were assessed with a pooled specificity with 95 % CIs.

### Statistical analysis

Outcome variables measured at specific time points were compared in terms of mean differences with 95 % CIs using a network meta-analysis. Analyses were based on non-informative priors for effect sizes and precision. Convergence and lack of auto-correlation were confirmed after four chains and a 50,000-simulation burn-in phase; finally, direct probability statements were derived from an additional 100,000-simulation phase. The probability that each group had the lowest rate of clinical events was assessed by Bayesian Markov Chain Monte Carlo modeling. Sensitivity analyses were performed by repeating the main computations with a fixed-effect method. Model fit was appraised by computing and comparing estimates for deviance and deviance information criterion. All statistical analyses were performed with Review Manager 5 and R (R version 3.0.3, R Foundation for Statistical Computing, Vienna, Austria; http://www.r-project.org) and the metafor and gemtc packages for pair-wise and network meta-analyses.

## Results

### Eligible studies

**Fig. 1:**
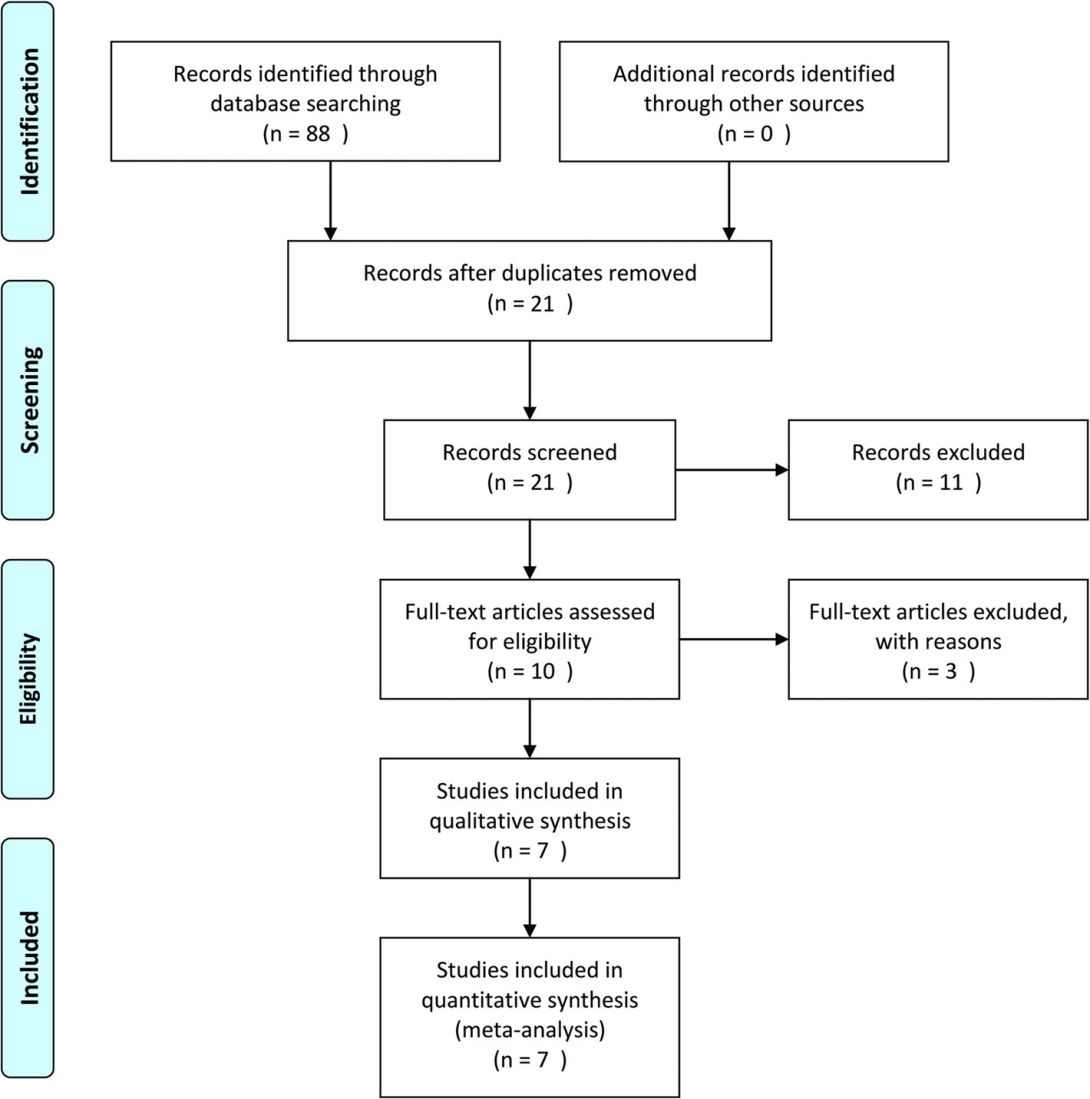
Flow diagram of evidence acquisition. Seven studies were ultimately included in the qualitative and quantitative syntheses using pairwise and network meta-analyses

The database search retrieved 21 articles covering 88 studies for potential inclusion in the meta-analysis. Fourteen articles were excluded based on the inclusion/exclusion criteria; eight of the fourteen articles were retrospective models, and three articles were reported with different tools and variables. The other three articles were excluded, because they did not report final results. Using using pairwise and network meta-analyses, the remaining seven articles were included in the qualitative and quantitative syntheses (Fig. 1).

Data corresponding to confounding factors in each study are summarized in Table 1. Four studies included outcome comparisons between alfuzosin versus placebo [15–18]. Two trials reported on therapeutic outcomes of tamsulosin versus placebo [19, 20], and a three-arm trial compared outcomes of alfuzosin, tamsulosin, and placebo [21].

**Table 1:** Enrolled studies for this meta-analysis

								Stent				Stone				
Study	Study design	N	Tx	P	Alpha-blocker	Analgesic	Duration (day)	Type	Size	Length	Indication	Size (mm)		Location	Measure	Quality assessment-risk bias
												Tx	P			
Beddingfield et al.[15]	RCT	55	26	29	Alfuzosin	On demand	10	NA	NA	Adjusted	Post URS	6.35	7.2	55 % renal	USSQ	Low
															- urinary symptom score	
					10 mg											
															- body pain score	
															- general health score	
														25 % renal/ureteric	- work performance score	
														20 % ureteric	- sexual health score	
Damiano et al.[20]	RCT	75	38	37	Tamsulosin	On demand	14	PU	7 Fr	Adjusted	Post URS	NA	NA	NA	USSQ	Intermediate
					0.4 mg										- urinary symptom score	
															- body pain score	
Deliveliotis et al.[16]	RCT	100	50	50	Alfuzosin	On demand	28	PU	5 Fr	Adjusted	Conservative treatment for stone, <10 mm, hydronephrosis	7.6	7.1	19 upper	USSQ	Low
														23 mid	- urinary symptom score	
					10 mg									48 lower	- body pain score	
															- general health score	
															- sexual health score	
Dellis et al.[21]	RCT	150	100	50*	Alfuzosin	On demand	28	NA	6 Fr	Adjusted	Post ESWL, URS	NA	NA	NA	USSQ	Low
															- urinary symptom score	
					10 mg											
					Tamsulosin										- body pain score	
					0.4 mg										- general health score	
															- work performance score	
															- sexual health score	
Nazim et al.[17]	RCT	130	65	65	Alfuzosin	On demand	7	PU	4.7 Fr 6 Fr	NA	Post URS	NA	NA	40 upper	VAS	High
														28 mid	USSQ	
					10 mg									62 lower	- urinary symptom score	
															- body pain score	
Park et al.[18]	RCT	32	20	12	Alfuzosin	NA	42	PU	6 Fr	Adjusted	Post URS, PCNL, Lap Pyelo, endo-ureterotomy	NA	NA	NA	USSQ	High
															- urinary symptom score	
															- body pain score	
					10 mg											
															- general health score	
															- work performance score	
															- sexual health score	
Wang et al.[19]	RCT	154	79	75	Tamsulosin	On demand	7	Sil	7 Fr	Adjusted	Post URS	9	9.4	16 upper	USSQ	Low
														49 mid	- urinary symptom score	
					0.4 mg									89 lower	- body pain score	
															- general health score	
															- work performance score	
															- sexual health score	

Adjusted, stent length is height adjusted; NA, not available; Tx, Treatment group, P, placebo; PU, Polyurethane; Sil, Silcone;USSQ, Ureteric Stent Symptom Questionnaire; VAS, visual analogue scale; URS, ureteroscopy; PCNL, percutaneous nephrolithotomy; Lap Pyelo, laparoscopic pyeloplasty

*50 patients received alfuzosin 10 mg, and another 50 patients received tamsulosin 0.4 mg

### Quality assessment

**Fig. 2:**
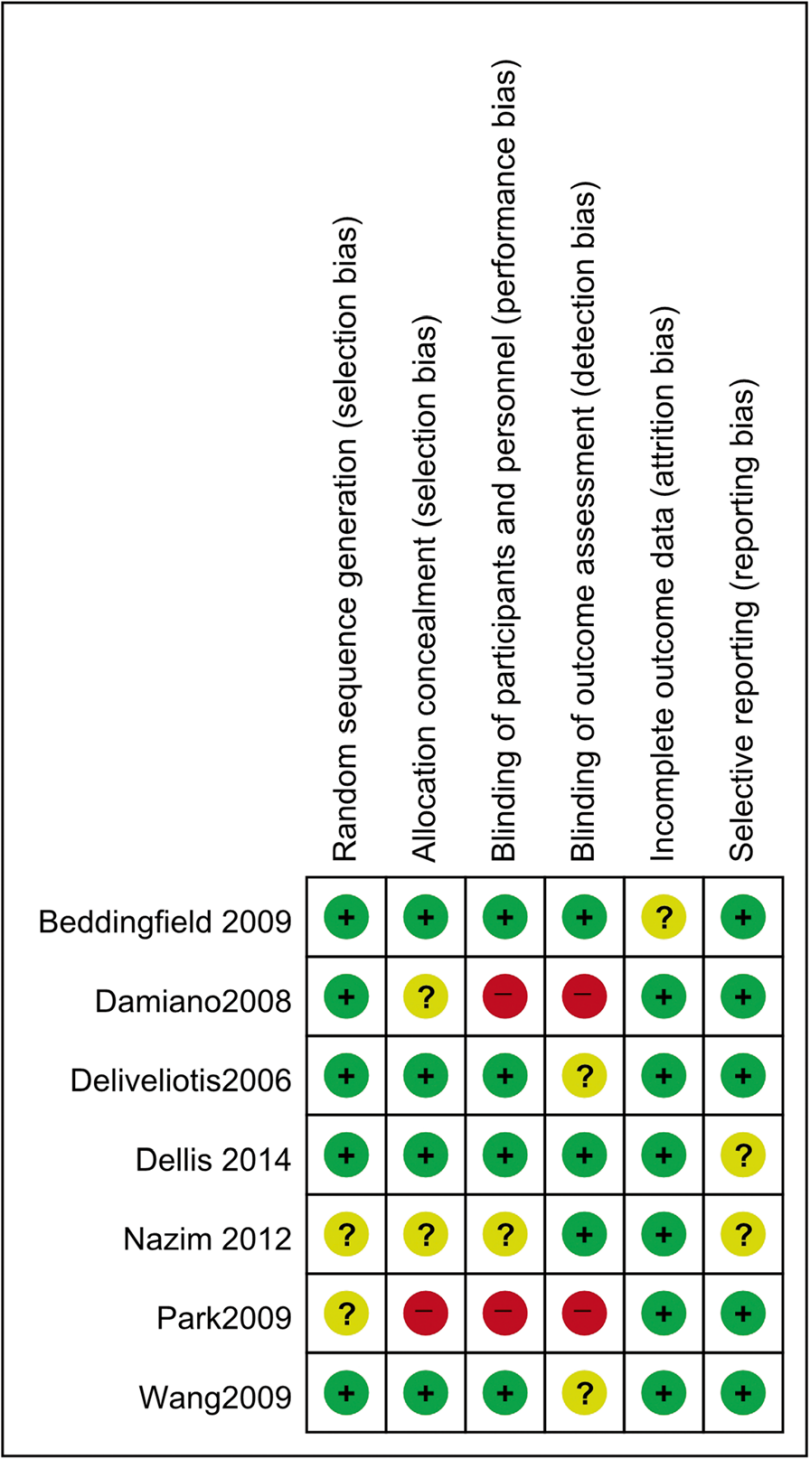
Risk of bias graph. We reviewed the risk of bias in each study included in this meta-analysis and presented the results as percentages. Four trials exhibited a low risk of bias for all quality criteria, and two studies were classified as having a high risk of bias

**Fig. 3:**
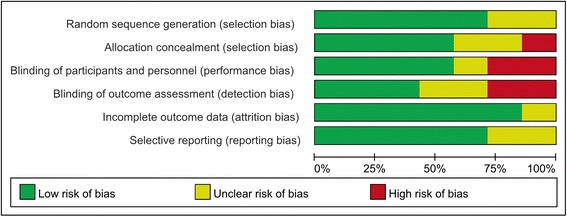
Risk of bias summary. We reviewed the risk of bias in each of the studies included in this meta-analysis

Figures 2 and 3 present the details of quality assessment, as measured by the Cochrane Collaboration risk-of-bias tool. Four trials exhibited a low risk of bias for all quality criteria, and two studies were classified as having a high risk of bias (Table 1). The most common risk factor for quality assessment was the risk of blinding of outcome assessment; the second most common concerns were allocation concealment and blinding participants and personnel.

### Heterogeneity assessment

**Fig. 4:**
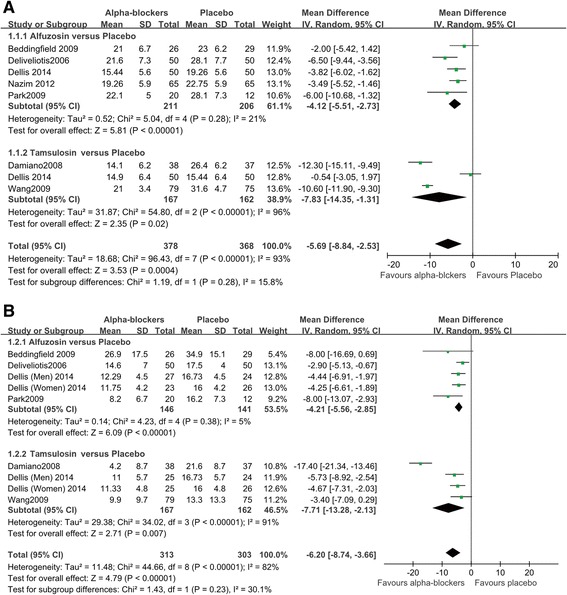
Pairwise meta-analysis. (**a**) urinary symptom score (**b**) body pain score

**Fig. 5:**
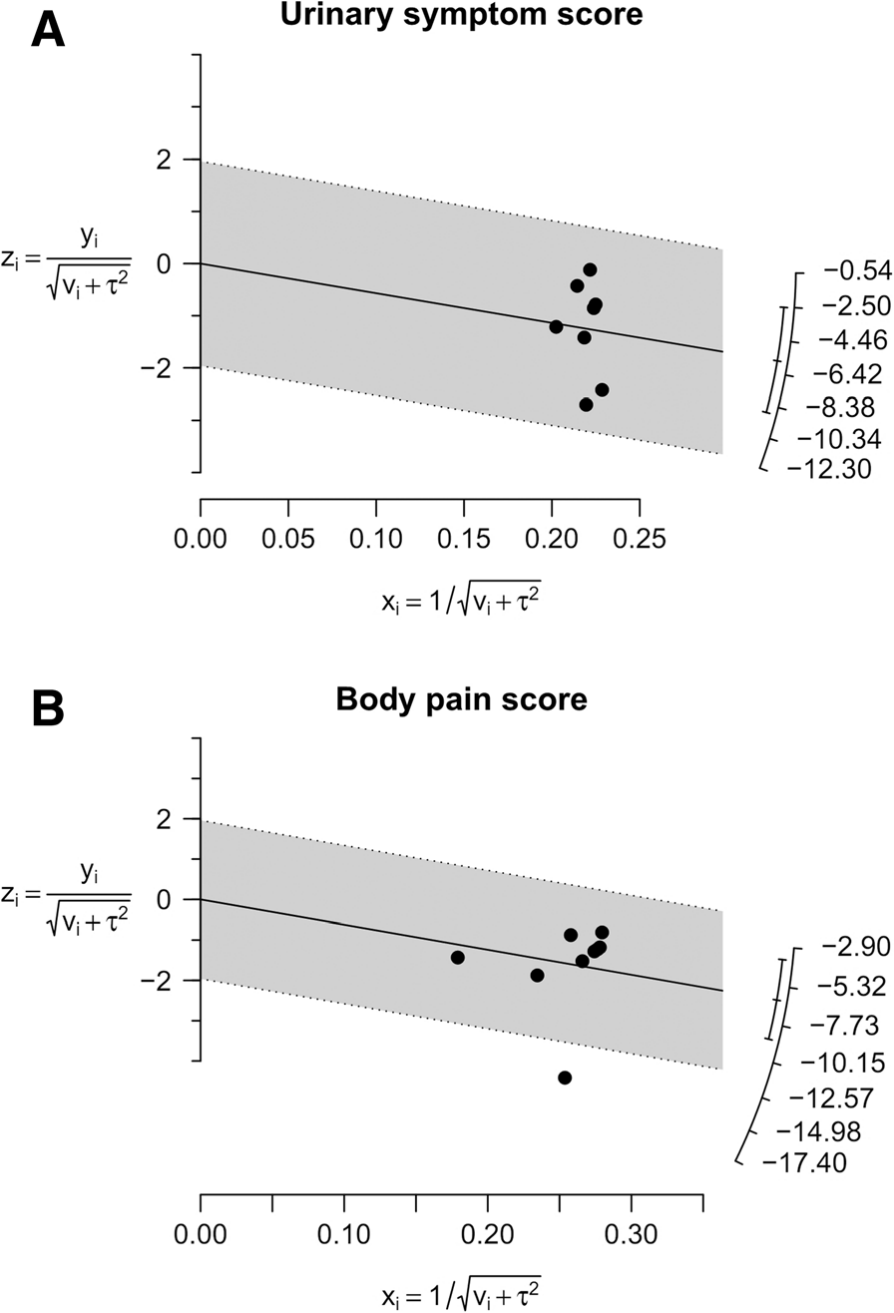
Radial plots. None of the variables demonstrated heterogeneity after selecting effect models for each variable in the radial plots. (**a**) urinary symptom score (**b**) body pain score

Forest plots of pairwise meta-analyses are demonstrated in Fig. 4. A heterogeneity test for USS showed the following: *χ*^2^ = 96.43 with 7 df (*P* < 0.001) and I^2^ = 93 % in the analysis of alpha-blockers, including alfuzosin and tamsulosin versus placebo. Notable heterogeneities were detected in the analyses of all studies; thus, random-effects models were used to further assess these variables. In the analysis of BPS, a heterogeneity test demonstrated homogeneity with *χ*^2^ = 44.66 with 8 df (*P* < 0.001) and I^2^ = 82 %. Pairwise meta-analyses with random-effects models were also performed. None of the variables demonstrated heterogeneity in radial plots after selecting effect models for USS and BPS (Fig. 5).

### Publication bias

**Fig. 6:**
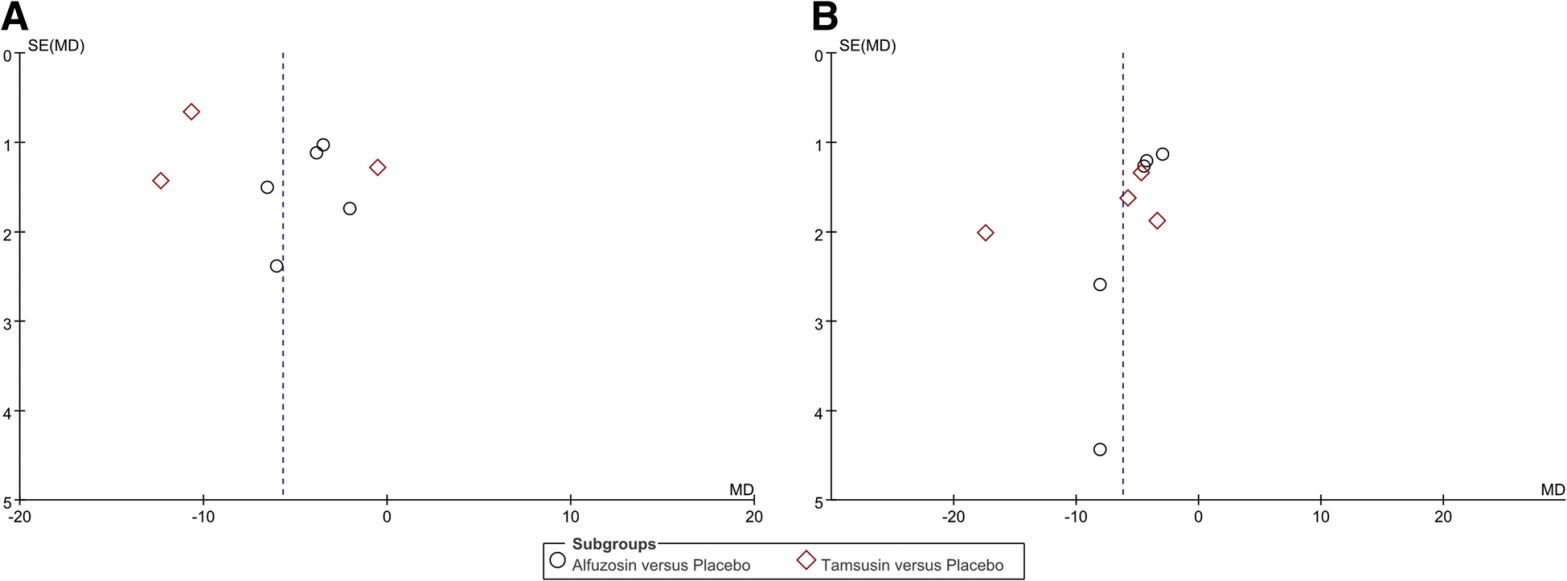
Funnel plots. (**a**) urinary symptom score (**b**) body pain score. It was difficult to assess publication bias with few studies, although some degree of bias is suspected

Funnel plots from pairwise meta-analyses are demonstrated in Fig. 6; however, with few studies, it was difficult to assess publication bias, although some degree of bias is suspected.

### Pairwise meta-analysis for urinary symptom and body pain scores

The forest plot using the random-effects model demomstrated an MD of −5.69 for USS (95 % CI [−8.84–−2.53], *P* < 0.001) between alpha-blockers and placebo (Fig. 4a). In subanalyses, both alfuzosin (MD; −4.12, 95 % CI [−5.51–−2.73], *P* < 0.001) and tamsulosin (MD; −7.83, 95 % CI [−14.35–−1.31], *P* = 0.02) had low MDs versus placebo. According to the forest plot for BPS, alpha-blockers were superior to placebo, with an MD of −6.20 (95 % CI [−8.74–−2.13], *P* < 0.001) (Fig. 4b). In the subanalysis of alfuzosin versus placebo, alfuzosin showed an MD of −4.21 (95 % CI [−5.56–−2.85], *P* < 0.001). Between tamsulosin and placebo, the random-effects model demonstrated an MD of −7.71 (95 % CI [−13.28–−2.13], *P* = 0.007) for BSP.

### Network meta-analysis for urinary symptom and body pain scores

**Fig. 7:**
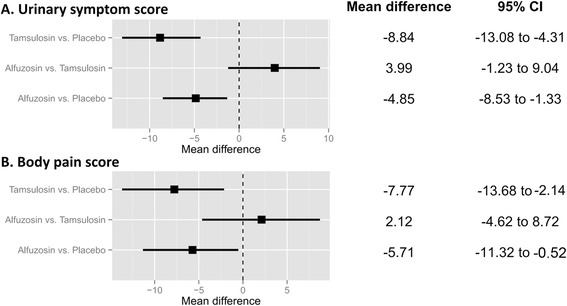
Network meta-analysis. (**a**) urinary symptom score (**b**) body pain score. Alfuzosin and tamsulosin had a lower score both in USS and BPS than in the placebo. However, there was not a significant difference in MD between alfuzosin and tamsulosin according to network meta-analysis

**Fig. 8:**
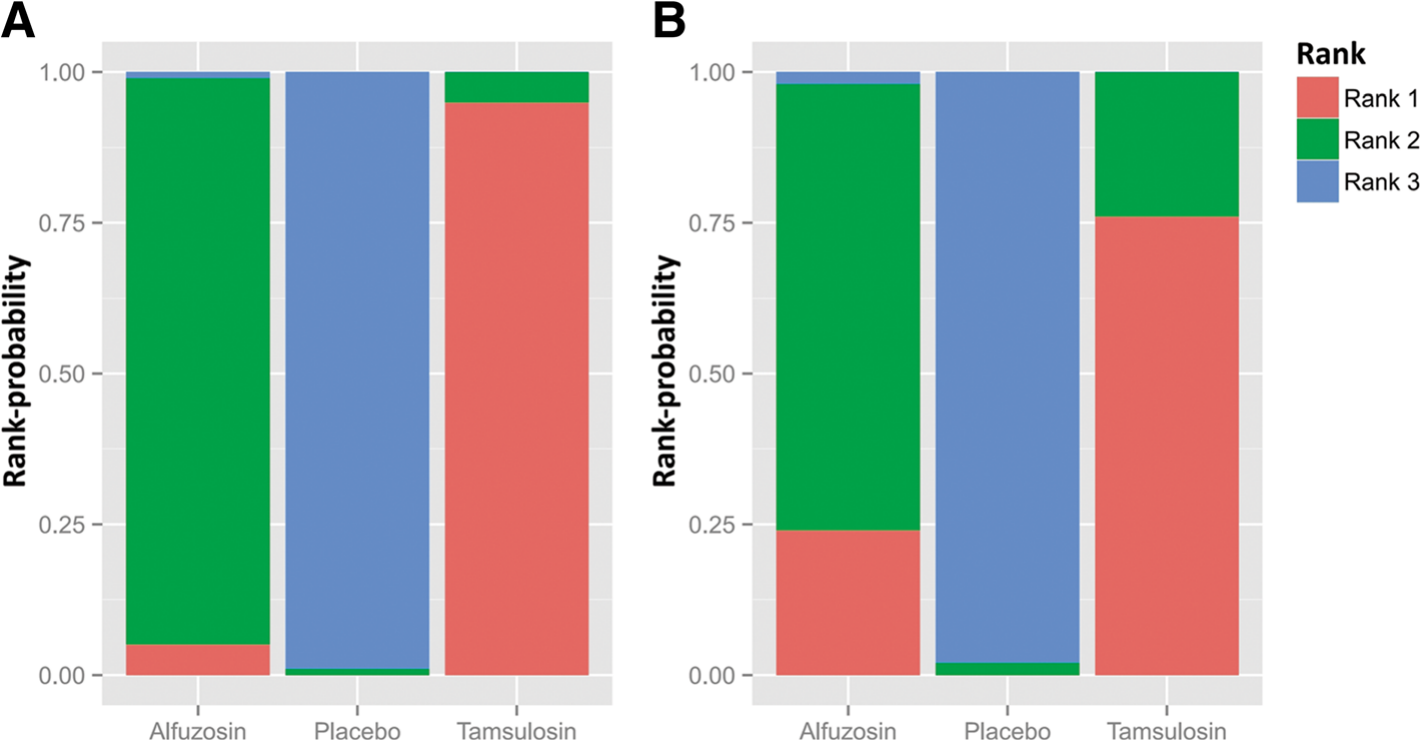
Rank-probability test of network meta-analyses. (**a**) urinary symptom score (**b**) body pain score. Tamsulosin had the highest rank for USS, followed by alfuzosin. Tamsulosin was also ranked highest for BPS in the rank-probability test, followed by alfuzosin. The placebo was ranked lowest for USS and BPS

Alfuzosin had a lower In USS than that of placebo (MD; −4.85, 95 % CI [−8.53–−1.33]). Tamsulosin also had a lower score than that of placebo (MD; −8.84, 95 % CI [−13.08–−4.31]. However, there was not a significant difference in MD between alfuzosin and tamsulosin according to network meta-analysis (MD; 3.99, 95 % CI [−1.23–9.04]). There were significant differences in the BPS achieved with alfuzosin versus placebo (MD; −5.71, 95 % CI [−11.32–−0.52]) and in tamsulosin versus placebo (MD; −7.77, 95 % CI [−13.68–−2.14]). Comparison of the BPS achieved with tamsulosin versus alfuzosin showed an MD of 2.12 (95 % CI [−4.62–8.72]), which was not significant (Fig. 7). In the rank-probability test, tamsulosin had the highest rank for USS, followed by alfuzosin. Tamsulosin was also ranked highest for BPS in the rank-probability test, followed by alfuzosin. The placebo was ranked lowest for USS and BPS (Fig. 8).

## Discussion and conclusion

Recently, two pair-wise meta-analyses of alpha-blockers in patients with ureteral stent discomfort were published. Yakoubi et al. conducted a meta-analysis of four RCTs [22]. However, they did not distinguish the types of alpha-blockers analyzed by each domain. Alpha-blockers reduced the scores for urinary symptoms, body pain, and general health index score but did not achieve significant changes in quality of work, sexual matters, or scores for additional problems. However, there were limits, as few studies were analyzed; in particular, only three studies were analyzed for quality of work and two for additional problems. Lamb et al. conducted a meta-analysis of five RCTs by distinguishing between alfuzosin and tamsulosin [7]; however, there was possible error in the effects-model with regards to heterogeneity. However, these meta-analyses showed decreased scores for both urinary symptoms and body pain after use of alpha-blockers.

More recently, Dellis et al. evaluated the effects of two different alpha-blockers for improving symptoms and quality of life in patients with indwelling ureteral stents in an RCT [21]. They prescribed alfuzosin, tamsulosin, or placebo to 50 patients and examined USSQ accordingly. Patients who received alpha-blockers had significantly decreased urinary symptoms, body pain, general health index, and sexual life scores compared to those of the control group. However, there was no difference in the quality of work score. Further, there was no difference between the alpha-blocker groups.

The goal of this study was to ascertain the difference between tamsulosin and alfuzosin in alleviating stent discomfort by comparing the effectiveness of alpha-blockers to placebo in seven RCTs based on urinary symptoms and body pain scores. In most studies, continuous treatment had been carried out before the removal of ureter stents. We divided alpha-blockers into alfuzosin and tamsulosin to conduct subgroup and network meta-analyses. In the subgroup analysis, two alpha-blockers appeared to significantly decrease urinary symptoms and body pain scores compared to those of the placebo. There was no statistical significance between alfuzosin and tamsulosin in network meta-analysis both in urinary symptoms and body pain scores, which was consistent with the results of previous studies. However, after conducting network meta-analysis using a rank-probability test, the effectiveness of tamsulosin appeared to be better than that of alfuzosin in both urinary symptoms and body pain scores (Fig. 8).

We suggested that the difference in efficacy between the two alpha-blockers arises from the physiologic characteristics of ureteral receptor distribution. The ureter contains two continuous thin muscle layers with a loosely spiraled internal layer and a more tightly spiraled external layer. A third outer longitudinal layer is located in the lower third of the ureter. The lower ureter is consisted of transitional epithelium, a connective tissue layer, and three layers of smooth muscle [23]. Peristalsis of ureter is initiated by spontaneous activity of the renal pelvis pacemaker cell and is essentially regulated by the myogenic mechanism and neurogenic factors; electrical and mechanical activities are conducted to inactive distal regions [24]. The histologic characteristics of the three smooth muscle layers in the lower portion of ureter and the denser innervation of the lower portion of ureter have become subjects of research interest. The alpha-1d receptor has the highest density in the lower portion of ureter. Tamsulosin is a subtype selective alpha-1a and alpha-1d blocker, whereas alfuzosin is a subtype non-selective alpha-1 blocker [25]. The two alpha-blockers may elicit different levels of efficacy due to differences in selectivity and the distribution of the alpha-1d receptor in the lower ureter. However, it remains unclear if subtype selectivity makes a significant contribution to the differences in efficacy of alpha-blockers. In the near future, prospective trials should compare several alpha-blockers, including silodosin and naftopidil, to confirm our results.

A limitation of our study was that only two subdomains were included, urinary symptoms and body pain scores, as not all the RCTs involved in this study had available USSQ domains besides urinary symptoms and body pain scores (Table 1). These may cause a bias in the efficacy of the two alpha-blockers, which can be influenced by other symptoms and quality of life. All RCTs included in the present study used USSQ, which comprehensively assesses not only urinary symptoms and pain, but also sexual symptoms and quality of life. Although urinary symptoms and pain are the most problematic among stent-related symptoms of discomfort, low abdominal pain or discomfort, infection, and hematuria are also bothersome to patients. Furthermore, alpha-blockers can cause side effects on the central nervous system, sexual function, ejaculatory function, and cardiovascular system [26]. Therefore, it is important to compare not only the efficacy between the two medications but also the side effects. In addition, comparison of USSQ domains may also need to be considered. Some degree of publication bias was also a limitation of this study. However, Sutton et al. reviewed 48 articles from the Cochrane Database of Systematic Reviews and showed publication or related biases were common within the sample of meta-analyses assessed [27]. Moreover, they found that these biases did not affect the conclusions in most cases. Another limitation was that we did not take into consideration the possible effects of stent factors on stent discomfort. Although six out of seven RCTs reported that stent insertion was performed with height adjustment and the differences of stent materials were also considered, there was no consideration as to whether the stent was placed correctly or not. Lee et al. [28]. reported that only storage symptoms of the tamsulosin group were significantly lower than those of the analgesic group in the appropriate stent position. However, this medication effect was not observed in the inappropriate stent position group, and total IPSS and storage symptom scores were significantly higher than in the appropriate stent position group. The appropriate stent position might be one of the points to be considered when conducting the research on stent discomfort.

## Conclusions

Ureteral stent-related symptoms are effectively alleviated with alpha-blockers. Tamsulosin might be more effective than alfuzosin. However, additional randomized controlled trials with alfuzosin and tamsulosin need to be performed for patients with ureteral stents.

## Abbreviations

RCTs: Randomized controlled trials
USSQ: Ureteric Stent Symptoms Questionnaire
USS: Urinary symptom score
BPS: Body pain score
